# A study on the turnover intention of teachers in Chinese regional universities: A predictive power analysis based on five management dimensions of high-performance human resource practices

**DOI:** 10.1371/journal.pone.0324487

**Published:** 2025-05-29

**Authors:** Jun Li, Lingjie Wang, Xiaoli Xu, Junyan Liu, Lei Peng

**Affiliations:** 1 School of Design, Hainan Vocational University of Science and Technology, Haikou, China; 2 Basic Course Department, Hengshui University, Hengshui, China; 3 Personnel Division, Hengshui University, Hengshui, China; 4 School of Economics and Management, Hengshui University, Hengshui, China; 5 School of Physical Education, Hengshui University, Hengshui, China; Universidade Europeia, Lisboa, PORTUGAL

## Abstract

In this study, the direct influences of five management dimensions of high-performance human resource practices (HPHRP), i.e., talent selection and cultivation (TSC), career and job security (CJS), performance appraisal and compensation incentives (PACI), participative management (PM), and affective incentives (AI), on Chinese local university teachers’ turnover intentions (TI) were investigated. Additionally, the mediating role of organizational commitment (OC) in these relationships was examined. According to the results of a questionnaire survey among 740 teachers from five regional colleges and universities in China, the five management dimensions of HPHRP significantly and negatively impact college teachers’ TI, with TSC being the most influential predictor (B = -0.359, t = -10.597***, R² = 0.132), followed by AI ( = -0.277, t = -7.829***, R² = 0.077), PACI ( = -0.262, t = -7.390***, R² = 0.069), CJS ( = -0.262, t = -7.390***, R² = 0.069), and PM ( = -0.231, t = -6.456***, R² = 0.053). Furthermore, OC was found to partially mediate the relationship between TSC (B = -0.243, t = -5.987***, R² = 0.164), CJS (B = -0.105, t = -2.621**, R² = 0.131), PACI (B = -0.127, t = -3.290**, R² = 0.136), and AI (B = -0.126, t = -3.125**, R² = 0.135) on college teachers’ TI and completely mediated the influence of PM (B = -0.053, t = -1.288, R² = 0.125) on college teachers’ TI. The findings suggest that TSC, OC, and PM play more significant roles in affecting Chinese regional university teachers’ TI. In order to reduce teachers’ TI, local university administrators should focus on talent, encourage teachers to take part in school management, and strengthen their commitment to the university.

## 1. Introduction

Chinese colleges and universities are classified into two categories based on their management attributes: state-affiliated and regional colleges and universities [1]. Within the Double First-Class Initiative framework in Chinese higher education, which aims to enhance the development of top-tier universities and disciplines, the current distribution of colleges and universities has been reshaped to some extent. However, the battle for talent snatching among universities has also been intensified. Impacted by the “siphon effect” caused by state-affiliated universities, regional colleges are at a disadvantage in the competition for better education resources and talents, aggravating teacher turnover [2]. In the long run, a moderate turnover rate facilitates a rational flow of talent, but a high turnover rate results in the loss of talent and disrupts the process of nurturing future talents [3]. Furthermore, the turnover of college teachers not only has a negative impact on the quality of teaching and student performance in the school, but it can also result in the discontinuation of important research projects, contribute to the uneven development of disciplines, and potentially compromise the availability of related courses [4,5]. Hence, it is of great significance to investigate the elements that influence regional college teachers’ turnover intentions (TI).

According to social exchange theory, the relationship between employees and organizations is based on reciprocal exchange, which means that when employees perceive the organization’s input, they will return the favor by acting in ways that will keep the reciprocal relationship going [6]. High-performance human resource practices (HPHRP) represent the organization’s investment in its employees, which communicates to employees that the organization values and cares about them and is willing to invest many resources in their professional growth; at this point, employees will report back to the organization with greater loyalty [7]. Prior research has demonstrated that the implementation of HPHRP plays a crucial role in decreasing employee turnover. For instance, Dorta-Afonso et al.(2023) [8] contended that when an organization adopted such practices, employees perceived that the organization valued their personal growth; consequently, they exhibited a favorable work attitude and were more inclined to remain with the organization as a form of reciprocation. Bochoridou and Gkorezis (2024) [9] discovered that implementing HPHRP in colleges and universities could predict a decreased likelihood of employees’ TI. Furthermore, research has demonstrated that faculty organizational commitment (OC) has a notable negative impact on faculty TI [10,11]. Therefore, this study employed HPHRP as the independent variable and OC as the mediator variable to explore the influence mechanism of teachers’ TI in regional colleges and universities in China.

Previous studies have verified the influence of HPHRP on employees’ TI in Western countries [9,12]. However, few studies have been conducted in Chinese cultural settings, especially in higher education organizations. Research has shown that Western culture emphasizes individualism and focuses on positive incentives like encouragement and employee rewards. In contrast, Chinese culture prioritizes collectivism and emphasizes positive and negative incentives like constraints and punishments, which aligns more with the multiple characteristics of HPHRP [13]. Additionally, past research has indicated that HPHRP is particularly well-suited for knowledge-based organizations [14,15]. Therefore, this study attempts to explore the use of HPHRP in Chinese higher education organizations to address the limitations of previous research. More importantly, previous research usually considered HPHRP as a single variable, neglecting the potential differences in the role of various management dimensions across different types of organizations. For instance, college teachers, who are knowledge-based employees, evaluate the significance of their work distinctly compared to employees in other organizations. Their primary need is to achieve self-worth realization, and they prioritize personal development and recognition of their value [14]. This study aimed to conduct a deeper investigation into the influence of different dimensions of HPHRP on college teachers’ TI to fill the gap in previous research.

Based on the aforementioned literature, while studies have established the negative predictive effect of HPHRP on employees’ TI in Western countries, it remains to be further verified whether it has the same effect in Chinese university organizations. Furthermore, it is unclear how the different dimensions of HPHRP impact college teachers’ TI. The primary goal of this research is to look into the impact of HPHRP on teachers’ TI at local Chinese institutions, as well as the predictive value of each management dimension on their TI. In order to accomplish this primary goal, this study establishes two secondary targets: first, it evaluates the impact of each management dimension on TI by reviewing the existing literature to gain a better understanding of the mechanisms and factors influencing the propensity to leave; second, it will use hierarchical regression analysis to test the predictive power of each management dimension on TI to validate the hypotheses and offer managerial recommendations.

This study holds considerable importance as it offers a theoretical framework and practical insights aimed at enhancing human resource management, addressing the pressing issue of teachers’ TI in regional colleges and universities, and ensuring the stability of the teaching faculty. Compared with the past studies, this study has two innovations: First, it applies HPHRP, which originated from enterprise organizations in Western countries, to the field of Chinese higher education and preliminarily verifies its applicability to the organizations of Chinese universities, thus realizing the interdisciplinary application of management theories in the field of education. Second, it analyzes the predictive power of the five dimensions of HPHRP on teachers’ TI, providing a more accurate theoretical basis for regional colleges and universities to reduce teachers’ TI.

The structure of this paper is organized as follows: the first part argues the research background, content, novelty, objectives, and significance; the second part analyzes the influential relationship among variables by reviewing the related literature and proposes the research hypotheses; the third part specifies the research design, sampling frame, sampling strategy, sample distribution, measurement tools and estimation techniques; the fourth part presents the data analysis process and presents the findings; the fifth part discusses the consistency and discrepancy of our findings with the existing literature and provides an analysis of the reasons; the sixth part summarizes the theoretical contributions, practical contributions, and policy implications; the seventh part presents the conclusions of the study; and, finally, the eighth part presents suggestions for future research in light of the limitations of this study.

## 2. Literature review and research hypotheses

### 2.1. HPHRP

Human resource practices have evolved from single-dimensional to multidimensional combinations. Early studies focus solely on the effects of staffing practices on organizational and employee performance [16]. However, later studies have shown that single-dimensional human resource practices have a limited impact on organizational performance. Human resource practices are primarily found in multidimensional combinations, such as talent selection and training, career and job guarantee, performance appraisal, advice in decision-making, and career development. These multidimensional combinations are known as HPHRP [17]. HPHRP in colleges and universities refers to the implementation of effective human resource management strategies within educational institutions. As categorized by Rao (2010) [18], HPHRP encompasses five key dimensions: talent selection and cultivation (TSC), career and job security (CJS), performance appraisal and compensation incentives (PACI), participative management (PM), and affective incentives (AI). TSC pertains explicitly to teachers’ promotion and further training, which plays a significant role in their personal development. CJS pertains to the university providing the necessary measures to ensure the stability of teachers’ work and scientific research. PACI primarily focuses on the effectiveness of the reward, assessment, and evaluation system, as well as the fairness of the process. PM refers to teachers’ involvement in decisions related to university management. AI refers to the provision of humanistic care and support for instructors. Considering the applicability to higher education organization settings, this study examines the five dimensions Rao (2010) [18] suggested and their impact on college teachers’ TI.

### 2.2. TSC, CJS, PACI, PM, AI, and TI

Previous studies have shown that a scientific and reasonable talent selection mechanism can ensure the introduction of teachers who are suitable for the development of the school; at the same time, a sound cultivation system can help teachers continuously improve their professionalism and teaching ability, enhance their sense of belonging to the profession, and reduce their tendency to leave [19,20]. CJS is crucial and significantly affects teachers’ TI since a good and stable working environment can enhance teachers’ sense of job security [21]. PACI can affect teachers’ motivation; a fair and seasonable PACI system can stimulate teachers’ enthusiasm for their work and effectively reduce teacher turnover [22]. PM gives employees more substantial discourse power and enables them to participate in organizational decision-making, enhancing their sense of ownership and thus playing an important role in reducing talent loss [12]. AI reflects the organization’s support, care, and recognition of employees, and emotional motivation creates a good organizational climate that can significantly reduce the tendency of employees to leave [23]. Therefore, this study proposes the hypothesis:

H1: TSC has a significant negative effect on TI;
H2: CJS has a significant negative effect on TI;
H3: PACI has a significant negative effect on TI;
H4: PM has a significant negative effect on TI;
H5: AI has a significant negative effect on TI;

### 2.3. The mediation of OC

#### 2.3.1. TSC, CJS, PACI, PM, AI, and OC.

A sound TSC system provides teachers with continuous learning and growth opportunities, such as professional training and academic exchanges, which will enhance their sense of identity and belonging to the organization and thus enhance OC [24,25]. CJS can provide employees with a stable working environment, which is crucial to the formation of OC, which has been found to enhance employees’ sense of job safety and reduce job burnout to promote the formation of a high level of OC [26]. Fair, reasonable, and motivational PACI mechanisms, which accurately measure employees’ work outcomes and motivate them to be more engaged in their work, positively impact employee OC [27]. Aiming to involve employees in the management decisions of the organization entirely, PM empowers teachers to make decisions in school affairs and makes them feel important and valued, which serves as an important measure to develop OC [28]. AI can create a warm and harmonious organizational atmosphere, enhance teachers’ emotional attachment, and play an important role in enhancing teachers’ OC [29].

#### 2.3.2. OC and TI.

Previous research has determined that OC measures employees’ overall perceptions of the organization they are employed in and significantly impacts their TI [30]. Meyer and Allen (1991) [31] argued that OC was primarily demonstrated through a strong sense of identification with the organization and a consequent feeling of responsibility, leading to a commitment to serve the organization for an extended duration and a reluctance to depart. Teacher’s OC is crucial in reducing teacher turnover in colleges and universities. Teachers with a strong sense of OC are emotionally and morally connected to their colleges and universities and are less likely to leave them [32]; thus, they are more likely to be loyal to their positions [33]. Empirical studies have also shown that teacher’ OC can negatively affect their TI [11,34].

**Fig 1:**
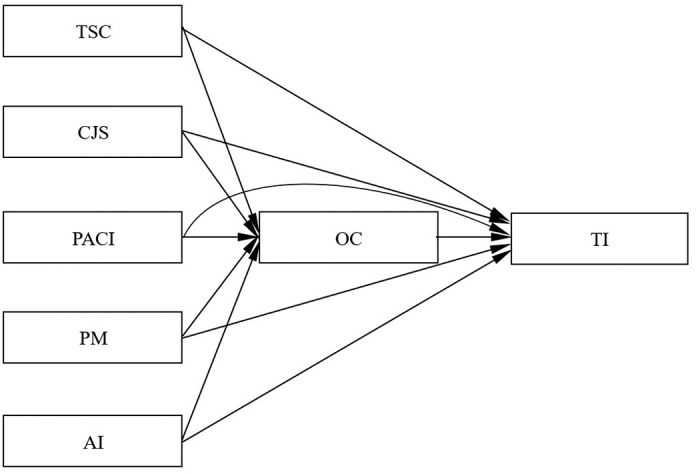
Conceptual model.

Based on the discussion above, HPHRP in colleges and universities facilitates teachers in aligning their values with the development goals and values of the institution. This alignment enhances teachers’ OC and willingness to remain employed by the university, thereby reducing their TI. Thus, our study suggests that the level of teachers’ OC may play a significant role in mediating the relationship between HPHRP and TI. Thus, the following hypotheses are suggested (the conceptual model is depicted in Fig 1):

H6: OC mediates the negative effect of TSC on TI;
H7: OC mediates the negative effect of CJS on TI
H8: OC mediates the negative effect of PACI on TI;
H9: OC mediates the negative effect of PM on TI;
H10: OC mediates the negative effect of AI on TI.

## 3. Research methodology

### 3.1. Research design and Sampling frame

The participants in this study are from five regional colleges and universities in Hebei Province, China. Hebei Province is geographically adjacent to Beijing, Tianjin, and other regions where many state-affiliated universities are located. Therefore, Hebei Province faces significant challenges in talent snatching due to the “siphon effect” caused by nearby regions like Beijing and Tianjin. As Xue and Li (2023) [35] described, this phenomenon negatively impacts the development of higher education in Hebei Province. Therefore, selecting college teachers from Hebei Province as a sample ensures higher representativeness. Given that the influence of Beijing and Tianjin on the talent pool in Hebei Province is limited by geographical distance, this study focused on five cities within Hebei that are adjacent to and border Beijing and Tianjin. Consequently, teachers from municipal undergraduate universities in these five cities were selected for sampling.

### 3.2. Sampling strategy

This study was approved by the Academic Ethics Committee of Hainan Vocational University of Science and Technology (HKD-2023–39) on April 15, 2023, and obtained participants’ written informed consent. According to James and Busher (2007) [36], this study’s data collection and analysis process was conducted anonymously to ensure the data’s authenticity and validity. In May 2023, 850 questionnaires were distributed through the online data platform. After excluding invalid questionnaires with the consistency of filling in the answers, responding with omissions questions, or those with too short a response time, 740 valid questionnaires were obtained with a return rate of 87.1%. According to Cappelleri et al. (2014) [37], the sample size criterion of a scale-involved survey should be at least 10 times the total number of scale questions, which was 52 in this study. Therefore, 740 valid samples in this study satisfied the minimum sample size condition. The demographic breakdown of the college teachers involved in the study was as follows: there were 356 male teachers (48.11%) and 384 female teachers (51.89%); additionally, there were 155 teachers with an undergraduate certificate (20.95%), 410 teachers with a master’s degree (55.41%), and 175 teachers with a PhD (23.65%).

### 3.3. Measures

#### 3.3.1. HPHRP scale.

The HPHRP scale for colleges and universities [38] was employed in this study. The scale was scored on a 5-point Likert scale, where a score of 1 represented totally inaccurate and a score of 5 represented totally accurate. Higher scores indicated higher levels of HPHRP among teachers. The scale included five subscales that measure five management dimensions: TSC, CJS, PACI, PM, and AI. The reliability analysis in this study revealed that the Cronbach’s α values for the five subscales were 0.944, 0.962, 0.965, 0.970, and 0.914, respectively. These values exceeded 0.700, indicating the scale’s good reliability [39]. The confirmatory factor analysis (CFA) results indicated that the RMR values for the five subscales were 0.049, 0.007, 0.029, 0.008, and 0.029, respectively, satisfying the criterion below 0.080. The NFI values were 0.878, 0.996, 0.942, 0.991, and 0.933; the IFI values were 0.881, 0.997, 0.944, 0.991, and 0.935; the TLI values were 0.834, 0.990, 0.916, 0.974, and 0.870; the CFI values were 0.881, 0.997, 0.944, 0.991, and 0.935; all of these values meeting the criterion of above 0.800. These results indicated that the model fit was acceptable [40]. Furthermore, the five subscales had composite reliability (CR) values of 0.944, 0.962, 0.965, 0.970, and 0.914, all exceeding 0.600. The average variance extracted (AVE) values were 0.680, 0.862, 0.798, 0.890, and 0.682, all exceeding 0.400. These results indicated good convergent validity [41].

#### 3.3.2. OC scale.

This study employed the OC Scale devised by Meyer et al. (1993) [42], which comprised three dimensions: affective, normative, and continuance commitment, with 18 question items. Affective commitment is the emotional connection between the employee and the organization and their identification with the organization. Normative commitment is devotion to correspond to the organization. Continuance commitment is the worker’s consciousness that they will lose out if they leave the organization [43]. The measure was assessed using a 5-point Likert scale, where a score of 1 represented totally inaccurate and a score of 5 represented totally accurate. Higher scores on the scale indicated more significant levels of OC. The reliability analysis in this study revealed a Cronbach’s α value of 0.927 for the scale, surpassing the threshold of 0.700 and indicating that the scale exhibited strong reliability [39]. The CFA results indicated that the GFI, NFI, IFI, TLI, and CFI values were 0.860, 0.862, 0.870, 0.849, and 0.870, respectively. These values exceeded the threshold of 0.800, demonstrating that the model fit fulfilled the acceptable standard [40]. Furthermore, the range of CR values was from 0.783 to 0.840, exceeding the threshold of 0.600. Similarly, the range of AVE values was from 0.443 to 0.478, exceeding the threshold of 0.400. These results indicated an ideal convergent validity [41].

#### 3.3.3. TI scale.

This study utilized the TI scale established by Cheng et al. (2015) [44], which consisted of two dimensions: resignation intentions and turnover plan. The scale included 6 question items and was assessed using a 7-point Likert scale, where a score of 1 represented totally inaccurate and a score of 7 represented totally accurate. Higher scores indicated a stronger TI. The reliability analysis in this study revealed that the Cronbach’s α value of the scale was 0.924, surpassing the threshold of 0.700 and indicating that the scale demonstrated good reliability [39]. The CFA yielded GFI = 0.934, AGFI = 0.827, NFI = 0.956, IFI = 0.958, TLI = 0.921, and CFI = 0.958, implying a good model fit [40]. Furthermore, the range of CR values was between 0.666 and 0.680, surpassing the threshold of 0.600. The range of AVE values was between 0.406 and 0.411, surpassing the threshold of 0.400. These results indicated a good convergent validity [41].

### 3.4. Estimation techniques

The data analysis process involved utilizing SPSS 21.0 and AMOS 25.0 in the subsequent stages. The first step involved testing the reliability and validity of the measurement instruments used in this study through reliability analysis and CFA. The second step involved conducting a common method variance (CMV) test using Harman’s one-factor test. The third step involved analyzing the participants’ overall performance on each variable and determining the degree of correlation between the variables through descriptive and Pearson correlation analyses. Lastly, a hierarchical regression analysis was conducted using SPSS 21.0 to examine the direct effect of each of the five management dimensions of HPHRP on TI. Additionally, the mediating role of OC in these relationships was also assessed. Ultimately, AMOS 25.0 was utilized to build a structural equation model to test whether the partial mediation model derived from the conclusions of this study has better explanatory power than the complete mediation model.

## 4. Results

### 4.1. CMV test

This study conducted a CMV test using Harman’s one-factor test with unrotated principal component factor analysis of all variable items. The analysis yielded five factors, each with an eigenroot greater than 1. The first factor accounted for 41.239% of the variance, below the critical criterion value of 50%. This finding indicated that no significant CMV issues were observed in this study [45].

### 4.2. Descriptive statistics and correlation analysis

Table 1 shows the descriptive statistics and correlation analysis of the seven variables: TSC, CJS, PACI, PM, AI, OC, and TI. The correlation coefficients between each variable pairwise ranged from -0.336 to -0.698, indicating that all the variables were moderately low correlated with each other and had no severe collinearity problems [46].

**Table 1:** Summary table of descriptive statistics and correlation analysis.

Variables	Mean Values	Standard Deviation	1	2	3	4	5	6	7
**TSC**	3.476	0.810	1						
**CJS**	3.074	1.003	0.663^***^	1					
**PACI**	3.346	0.946	0.634^***^	0.698^***^	1				
**PM**	3.444	0.990	0.525^***^	0.589^***^	0.443^***^	1			
**AI**	3.373	0.771	0.698^***^	0.636^***^	0.551^***^	0.591^***^	1		
**OC**	3.741	0.621	0.559^***^	0.509^***^	0.464^***^	0.554^***^	0.531^***^	1	
**TI**	2.523	1.194	-0.363^***^	-0.256^***^	-0.262^***^	-0.231^***^	-0.277^***^	-0.351^***^	1

*means p < 0.05,

**means p < 0.01,

***means p < 0.001, the same belo

### 4.3. Regression analysis of five management dimensions of HPHRP, OC, and TI

**Fig 2:**
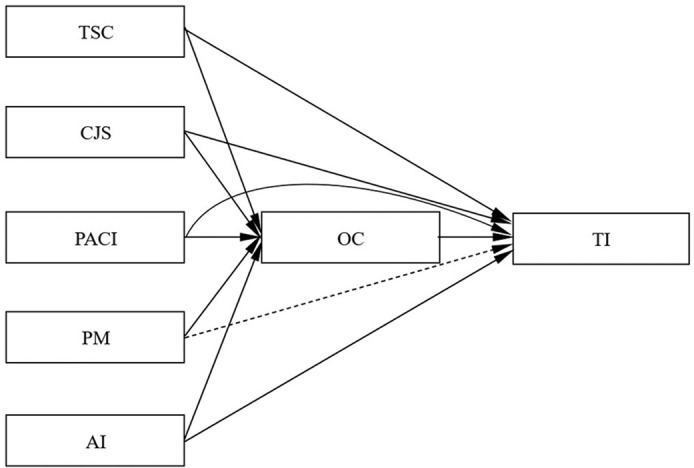
The effect plots for each path. (a) A solid line indicates a significant path effect. (b) A dashed line indicates a non-significant path effect.

This study employed regression analysis to investigate the impact of five management dimensions of HPHRP (TSC, CJS, PACI, PM, AI) on OC and TI, respectively. Additionally, the study examined the influence of OC on TI was also examined. Fig 2 presents the effect plots for each path. As indicated in Table 2, the data results demonstrated that all five management aspects of HPHRP exerted a substantial negative impact on TI in Model 1. The analysis revealed that TSC had the highest predictive power (B = -0.359, t = -10.597***, R² = 0.132) in determining TI, followed by AI ( = -0.277, t = -7.829***, R² = 0.077), PACI ( = -0.262, t = -7.390***, R² = 0.069), CJS ( = -0.262, t = -7.390***, R² = 0.069), and PM ( = -0.231, t = -6.456***, R² = 0.053). The results underscored the negative impact of these management dimensions on TI, with TSC having the highest predictive power and PM the weakest.

**Table 2:** Regression analysis of 5 management dimensions of HPHRP, OC and TI.

	Model 1			Model 2			Model 3		
	TI			OC			TI		
	(*t*)	R²	F	(*t*)	R²	F	(*t*)	R²	F
**TSC**	-0.359(-10.597***)	0.132	112.293***	0.559(18.331***)	0.313	336.043***	-0.243(-5.987***)	0.164	72.151***
**OC**							-0.215(-5.283***)		
**CJS**	-0.256(-7.198***)	0.066	51.818***	0.509(16.080***)	0.259	258.553***	-0.105(-2.621**)	0.131	55.629***
**OC**							-0.298(-7.457***)		
**PACI**	-0.262(-7.390***)	0.069	54.615***	0.464(14.218***)	0.215	202.153***	-0.127(-3.290**)	0.136	57.884***
**OC**							-0.292(-7.550***)		
**PM**	-0.231(-6.456***)	0.053	41.678***	0.554(18.064***)	0.307	326.319***	-0.053(-1.288)	0.125	52.657***
**OC**							-0.321(-7.765***)		
**AI**	-0.277(-7.829***)	0.077	61.286***	0.531(17.077***)	0.282	289.236***	-0.126(-3.125**)	0.135	57.278***
**OC**							-0.284(-7.019***)		

In Model 2, all five management dimensions significantly positively impacted OC. Specifically, The analysis revealed that TSC (B = 0.559, t = 18.331***, R² = 0.313) had the highest predictive power in determining OC, followed by PM ( = 0.554, t = 18.064***, R² = 0.307), AI ( = 0.531, t = 17.077***, R² = 0.282), CJS ( = 0.509, t = 16.080***, R² = 0.259), and PACI ( = 0.464, t = 14.218***, R² = 0.215). The results revealed the negative impact of these management dimensions on OC, with TSC having the highest predictive power and PACI the weakest.

In model 3, it was revealed that TSC, CJS, PACI, and AI still significantly negatively impact TI after including OC as a mediating variable. However, their influence was reduced compared to model 1. On the other hand, the negative influence of PM on TI was no longer significant. It suggested that OC partially mediates the relationships between TSC, CJS, PACI, AI, and TI while completely mediating the relationship between PM and TI.

Moreover, upon conducting a more detailed analysis, it was evident that the inclusion of the mediating variable of OC affected the explanatory rates of five management dimensions of HPHRP on TI. Specifically, their explanatory rates were sorted as TSC (R² = 0.164)>PACI (R² = 0.136)>AI (R² = 0.135)>CJS (R² = 0.131)>PM (R² = 0.125). The results indicated that the factors of TSC and OC (R² = 0.164) had the highest rate of explaining TI.

### 4.4. Supplementary analysis-structural model comparison

**Fig 3:**
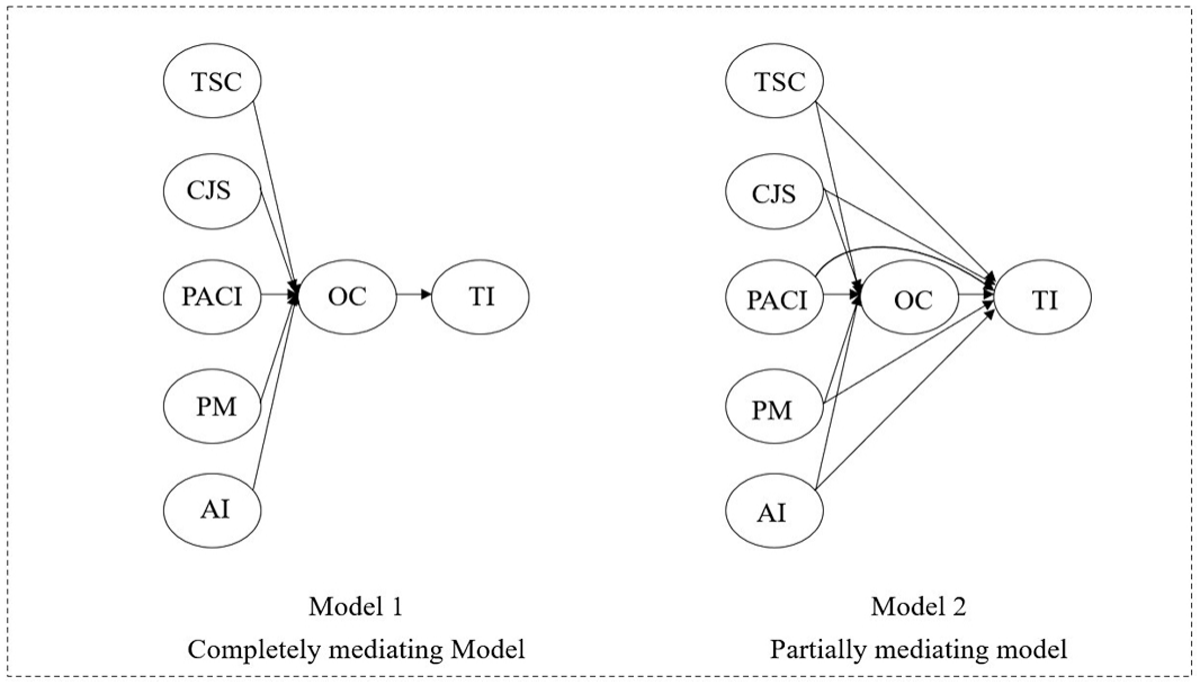
Models comparison.

Regression analyses showed that OC partially mediated the negative effects of TSC, CJS, PACI, and AI on TI, except for PM. In order to test whether the partial mediation model has better explanatory power for TI than a complete mediation model, AMOS 25.0 was used to compare the fitness of the two models (in Fig 3). Hu and Bentler (1998) [47] argued that a smaller value of ²/ df indicated a better fit of the overall model’s causal paths to the actual information. Hsiao et al. (2016) [40] further emphasized that RMR should be less than 0.08, with a smaller value indicating a better model fit; CFI, TLI, IFI, and NFI should be greater than 0.8, with larger values indicating a better model fit. Our findings, as shown in Table 3, confirmed the superiority of the partial mediation model. Both models fit at a reasonable level, but the partial mediation model outperforms the complete mediation model with smaller values of ²/ df and RMR and larger values of CFI, TLI, IFI, and NFI.

**Table 3:** Models fit comparison of the complete and partial mediation models.

	*x*²*/df*	RMR	CFI	TLI	IFI	NFI
Complete mediation model	8.575	0.048	0.871	0.858	0.872	0.857
Partial mediation model	8.560	0.044	0.873	0.859	0.873	0.859

## 5. Discussion

The regression analysis results reveal that TSC plays a more critical role in influencing TI than other management dimensions of HPHRP. This impact can occur directly or indirectly through the mediating role of OC. This result is consistent with the majority of past research, which has suggested that a well-developed TSC system enhances teacher-job fit and provides teachers with opportunities for continued professional growth, leading to teachers exhibiting higher levels of loyalty and a lower TI [19,20,48]. This result can be explained by the social exchange theory, in which universities invest in teachers through a well-developed TSC system, and teachers will return to universities with high OC and low TI levels [6]. In addition, the results also show some uniqueness while reinforcing existing views. For instance, Cao et al. (2013) [49] findings show that compensation levels play a more important role in influencing the propensity of employees to leave a firm than TSC. There are two possible reasons for this discrepancy: First, college teachers, as knowledge-based employees, tend to place greater emphasis on personal growth opportunities and the fulfillment of their potential compared to other types of employees [50]. If teachers’ personal development is restricted and they are unsatisfied with personal fulfillment, their TI will be heightened [51]. Second, the participants in this study were faculty members of regional colleges and universities in China, which are at a disadvantage in terms of talent management strategy and faculty development platforms relative to state-affiliated universities. Consequently, if colleges and universities fail to invest in TSC adequately, the teachers may resign if their personal development requirements are not fulfilled [52].

Regression analysis revealed that PM significantly and negatively impacted TI and positively affected OC, which is worthy of notice. However, when OC was considered a mediating variable, the direct effect of PM on TI became insignificant. Instead, the mediating effect of OC remained significant, suggesting that OC entirely mediated the influence of PM on TI. Few studies explore the relationship between the PM, TI, and OC variables. Some studies have discovered other mediators between PM and TI rather than OC. For example, Kumar & Jauhari’s (2016) [53]study showed that organizational justice and learning goal/need satisfaction mediated the relationship between employee engagement on TI. Fattah et al. (2022) [54] found that organizational support mediated the relationship between participative decision-making and TI. The results of past and present studies do not form an agreement, and this difference may be related to the characteristics of local colleges. Due to the small size of regional colleges and universities, the relatively fixed personnel has led to a close interpersonal network between teaching and administrative faculties. This close relationship is further strengthened and fully transformed into OC during participation in the management process, inhibiting TI. Employees in other organizations, especially in business, are highly mobile and unstable. Their emotional adhesion to the organization is relatively weak, which may lead to the limited enhancement of OC during participation in management. OC fails to form a mediating factor that adequately affects TI.

Furthermore, compared to other dimensions of HPHRP, PM exhibited the lowest level of predictive power in terms of its direct and indirect impact through OC on TI. However, it demonstrated a significant predictive power in influencing OC, ranking second only to TSC. These results imply that whereas PM has a limited ability to predict TI directly, it has a remarkable ability to predict the intermediary variable of OC. This result is similar to past studies. For example, Yandi and Havidz (2022)[28] showed a significant positive correlation between employee involvement in decision-making and OC; Ohana et al. (2013) [55]found that employee involvement had a significant positive effect on OC. It may be because PM facilitates effective communication between different levels of the organization, strengthens organizational cohesion, and aligns the organization’s development goals with the personal development goals of all employees. As a result, employees become more committed to the organization [56]. Furthermore, based on the social exchange theory, teachers commit greater effort to their responsibilities in exchange for their universities, thereby cultivating a robust OC.

## 6. Research contributions

### 6.1. Theoretical contributions

The theoretical contributions of this study lie in the following: First, this study expands the scope of HPHRP theory, which originated from business organizations in Western countries, by applying it to the research context of regional colleges and universities in China, providing an empirical basis for verifying the cross-cultural applicability and development of the theory. Second, it provides an in-depth analysis of the predictive power of the five dimensions of HPHRP on teachers’ TI, which makes up for the inadequacy of previous studies in precisely exploring the relationship between the two variables. Third, it integrates the theoretical strengths of management and education to provide a new perspective and comprehensive understanding of teachers’ TI in regional colleges and universities in China.

### 6.2. Practical contributions

The practical contributions of this study include the following: First, this study provides local university administrators with a precise direction for optimizing human resource management through an in-depth analysis of the predictive power of the HPHRP’s five management dimensions on teachers’ TI. Second, it reveals in-depth the influence mechanism of HPHRP on teachers’ TI, which can provide a reference for educational management departments to formulate policies on university faculty development, such as rational allocation of educational resources and particular policy inclinations for regional colleges and universities. Third, this study can provide practical references for local university administrators to reduce teachers’ TI.

### 6.3. Policy implication

According to the study results, TSC is the primary element influencing college teachers’ TI out of the five management dimensions of HPHRP. This finding suggests that regional colleges and universities should prioritize enhancing efforts toward TSC while implementing human resource management reform. For instance, universities should allocate adequate funds for teachers’ professional development in the annual budgets. Additionally, universities should design comprehensive plans for teachers’ further training and academic exchanges based on the development of academic disciplines and majors. Furthermore, universities could conduct thorough research and create specific talent team-building plans when designing development strategies. Maximize the effectiveness of the Teacher Development Center by helping each teacher create a personalized career development plan that aligns with their requirements.

Considering the significant influence of OC on teachers’ TI, colleges and universities must prioritize the development of teachers’ OC. Firstly, they can establish a highly effective and equitable interactive platform between colleges and teachers to strengthen their emotional bond and enhance teachers’ sense of responsibility towards the institution. Secondly, creating a favorable working environment and fostering a positive and vibrant atmosphere can help colleges and universities stay attuned to teachers’ psychological well-being and understand their emotional fluctuations towards the institution. If teachers experience negative emotional changes towards colleges and universities, it is crucial for them to promptly communicate with their colleagues to identify the underlying causes of the problem. Additionally, in order to cultivate an inspiring cultural atmosphere, it is crucial to promote the historical traditions and philosophy of the college, which will ensure that teachers’ values, ideals, and beliefs align with the college’s cultural environment and development principles, thereby enhancing their level of OC.

Even though both PM’s direct and indirect influences on TI appeared somewhat minimal, it is also crucial to highlight PM’s significance due to its strong effect on the mediating variable of OC, which can further impact TI. Therefore, colleges and universities should grant teachers more opportunities to participate in daily management to enhance their OC. First, enhancing the information communication between school leaders and teachers is crucial. It can be achieved by establishing a seamless feedback and complaint system where teachers’ opinions and suggestions on school management are thoroughly considered and promptly addressed. Second, when making important decisions regarding policy formulation, cadre appointments, assessment appraisal, and development planning, it is essential to seek extensive participation from teachers. Furthermore, when convening school leadership meetings to discuss significant matters, the college can invite teacher representatives to participate actively in implementing democratic centralism. These approaches can help foster a sense of ownership among the teachers and enhance their PM.

## 7. Conclusion

This study examined the direct effects of each of the five management dimensions of HPHRP (namely, TSC, CJS, PACI, PM, and AI) on TI, as well as the mediating effect of OC in these relationships by recruiting teachers from regional colleges and universities in China as participants. The following conclusions are drawn:

First, all five management dimensions of HPHRP can significantly and negatively affect teachers’ TI, with TSC having the most potent predictive power; Second, OC partially mediated between the effects of TSC, CJS, PACI, and AI on TI, respectively; Third, OC completely mediated the effects of PM on TI.

## 8. Limitations and future research directions

This study has several constraints. First, the data was obtained solely from self-reports provided by college teachers. Despite Harman’s one-way test indicating the absence of a significant CMV problem, it is recommended that future studies incorporate diverse research methods, such as interviews and observations, to mitigate the inevitable bias caused by the single research methodology. Second, this study relied on cross-sectional data, illustrating the association between each variable at a specific moment rather than establishing any cause-and-effect relationship between the variables. While some believe that cross-sectional studies can offer significant insights into the link between factors [57], future research may validate the findings of this study using longitudinal data. Third, this study was limited to the scope of local Chinese universities, which initially verified the positive effects of HPHRP in the Chinese cultural context and could not confirm the cross-cultural applicability of HPHRP theory. Future research could expand the scope of the study to validate the results further. Fourth, the results of this study only reflect the importance of five management dimensions of HPHRP (such as TSC and PM) in impacting faculty TI in local Chinese universities and did not address or compare the situation in state-affiliated universities. Future research could utilize the comparative research method to analyze the differences in the factors influencing teachers’ TI between state-affiliated and regional colleges and universities to gain a more comprehensive understanding of the deeper causes of the stability of the teaching force in different types of universities.

## Supporting information

S1 Data Original data. (XLSX)

S1 Check list STROBE statement–Checklist of items that should be included in reports of observational studies. (DOCX)
