# Supplementary material for: A study on the turnover intention of teachers in Chinese regional universities: A predictive power analysis based on five management dimensions of high-performance human resource practices
Source: PLoS One. 2025 May 29;20(5):e0324487. doi: 10.1371/journal.pone.0324487 (PMC12121734; doi:10.1371/journal.pone.0324487)
Supplement: S1 Check list — (DOCX) [file pone.0324487.s002.docx]

STROBE Statement—checklist of items that should be included in reports of observational studies

|  | Item No. | Recommendation | Page  No. | Relevant text from manuscript |
| --- | --- | --- | --- | --- |
| **Title and abstract** | 1 | (*a*) Indicate the study’s design with a commonly used term in the title or the abstract | 1 | The turnover intention of teachers in chinese regional universities: A predictive power analysis based on five management dimensions of high-performance human resource practices |
|  |  | (*b*) Provide in the abstract an informative and balanced summary of what was done and what was found | 2 | Background: The issue of brain drain in Chinese regional universities is escalating in severity. Research has demonstrated that high-performance human resource practices (HPHRP) significantly affect college teacher turnover intentions (TI). However, it remains uncertain which management dimension has a more pronounced impact on college teachers’ TI.  Methods: The present study examines the direct effects of five management dimensions of HPHRP, i.e., talent selection and cultivation (TSC), career and job security (CJS), performance appraisal and compensation incentives (PACI), participative management (PM), and affective incentives (AI) on TI among a sample of 740 teachers from five regional universities. Additionally, the role of organizational commitment (OC) as a mediator in these effects was explored.  Results: The findings indicate that the five management dimensions of HPHRP significantly and negatively impact college teachers’ TI, with TSC being the most influential predictor. Furthermore, the influence of TSC, CJS, PACI, and AI on college teachers’ TI was partially mediated by OC; OC completely mediated the influence of PM on college teachers’ TI.  Conclusion: The present study enhances comprehension of the mechanisms underlying the five management dimensions of HPHRP that impact OC and TI among college teachers. Additionally, it offers a more precise guideline for regional universities to mitigate college teachers’ TI. |
| Introduction | | | |  |
| Background/rationale | 2 | Explain the scientific background and rationale for the investigation being reported | 3-5 | as specific details in the manuscript Page 3-5 |
| Objectives | 3 | State specific objectives, including any prespecified hypotheses | 10-11 | Hypothesis 1 was proposed: TSC has a significant negative effect on TI;  Hypothesis 2 was proposed: CJS has a significant negative effect on TI;  Hypothesis 3 was proposed: PACI has a significant negative effect on TI;  Hypothesis 4 was proposed:  PM has a significant negative effect on TI;  Hypothesis 5 was proposed:  AI has a significant negative effect on TI;  Hypothesis 6 was proposed: OC mediates between the negative effect of TSC on TI;  Hypothesis 7 was proposed: OC mediates between the negative effect of CJS on TI  Hypothesis 8 was proposed: OC mediates between the negative effect of PACI on TI;  Hypothesis 9 was proposed: OC mediates between the negative effect of PM on TI;  Hypothesis 10 was proposed: OC mediates between the negative effect of AI on TI.  Concisely, this study sought to examine the following two inquiries:(1) Whether TSC, CJS, PACI, PM and AI affect the turnover intention of teachers in chinese regional universities and what is their predictive power? (2)Does OC play a mediating role in these relationships? |
| Methods | | | |  |
| Study design | 4 | Present key elements of study design early in the paper | 11-14 | as specific details in the manuscript Page 11-14 |
| Setting | 5 | Describe the setting, locations, and relevant dates, including periods of recruitment, exposure, follow-up, and data collection | 11 | as specific details in the manuscript Page 11 |
| Participants | 6 | (*a*) *Cohort study*—Give the eligibility criteria, and the sources and methods of selection of participants. Describe methods of follow-up  *Case-control study*—Give the eligibility criteria, and the sources and methods of case ascertainment and control selection. Give the rationale for the choice of cases and controls  *Cross-sectional study*—Give the eligibility criteria, and the sources and methods of selection of participants | 11 | This study employed convenience sampling to survey 740 teachers from five regional colleges in Hebei Province, China. Hebei Province is geographically adjacent to Beijing, Tianjin, and other regions where many state-affiliated universities are located. Therefore, Hebei Province faces significant challenges in talent snatching due to the “siphon effect” caused by nearby regions like Beijing and Tianjin. As Xue and Li (2023) [34] described, this phenomenon negatively impacts the development of higher education in Hebei Province. Therefore, selecting college teachers from Hebei Province as a sample ensures higher representativeness. |
|  |  | (*b*) *Cohort study*—For matched studies, give matching criteria and number of exposed and unexposed  *Case-control study*—For matched studies, give matching criteria and the number of controls per case |  |  |
| Variables | 7 | Clearly define all outcomes, exposures, predictors, potential confounders, and effect modifiers. Give diagnostic criteria, if applicable | 12-14 | as specific details in the manuscript Page 12-14 |
| Data sources/ measurement | 8* | For each variable of interest, give sources of data and details of methods of assessment (measurement). Describe comparability of assessment methods if there is more than one group | 12-14 | as specific details in the manuscript Page 12-14 |
| Bias | 9 | Describe any efforts to address potential sources of bias | 15 | This study conducted a CMV test using Harman’ s one-factor test with unrotated principal component factor analysis of all variable items. The analysis yielded five factors, each with an eigenroot greater than 1. The first factor accounted for 41.239% of the variance, below the critical criterion value of 50%. This finding indicated no significant CMV issues were observed in this study |
| Study size | 10 | Explain how the study size was arrived at | 11-12 | In May 2023, a total of 850 questionnaires were distributed through an online data platform in this study. After excluding invalid questionnaires, 740 formal questionnaires were obtained. The recovery rate was 87.1%. Participants completed the survey voluntarily. The data collection and analysis process was carried out in an anonymous manner. The demographic breakdown of the college teachers involved in the study was as follows: there were 356 male teachers (48.11%) and 384 female teachers (51.89%); additionally, there were 155 teachers with an undergraduate certificate (20.95%), 410 teachers with a master’s degree (55.41%), and 175 teachers with a PhD (23.65%). |

Continued on next page

| Quantitative variables | 11 | Explain how quantitative variables were handled in the analyses. If applicable, describe which groupings were chosen and why | 14 | Statistical analysis |
| --- | --- | --- | --- | --- |
| Statistical methods | 12 | (*a*) Describe all statistical methods, including those used to control for confounding | 14 | Statistical analysis |
|  |  | (*b*) Describe any methods used to examine subgroups and interactions |  |  |
|  |  | (*c*) Explain how missing data were addressed |  |  |
|  |  | (*d*) *Cohort study*—If applicable, explain how loss to follow-up was addressed  *Case-control study*—If applicable, explain how matching of cases and controls was addressed  *Cross-sectional study*—If applicable, describe analytical methods taking account of sampling strategy |  |  |
|  |  | (*e*) Describe any sensitivity analyses |  |  |
| Results | | | | |
| Participants | 13* | (a) Report numbers of individuals at each stage of study—eg numbers potentially eligible, examined for eligibility, confirmed eligible, included in the study, completing follow-up, and analysed | 11-12 | as specific details in the manuscript Page 11-12 |
|  |  | (b) Give reasons for non-participation at each stage |  | NA |
|  |  | (c) Consider use of a flow diagram |  | NA |
| Descriptive data | 14* | (a) Give characteristics of study participants (eg demographic, clinical, social) and information on exposures and potential confounders | 11-12 | as specific details in the manuscript Page 11-12 |
|  |  | (b) Indicate number of participants with missing data for each variable of interest |  |  |
|  |  | (c) *Cohort study*—Summarise follow-up time (eg, average and total amount) |  |  |
| Outcome data | 15* | *Cohort study*—Report numbers of outcome events or summary measures over time |  |  |
|  |  | *Case-control study—*Report numbers in each exposure category, or summary measures of exposure |  |  |
|  |  | *Cross-sectional study—*Report numbers of outcome events or summary measures | 16-18 | as specific details in the manuscript Page 16-18 |
| Main results | 16 | (*a*) Give unadjusted estimates and, if applicable, confounder-adjusted estimates and their precision (eg, 95% confidence interval). Make clear which confounders were adjusted for and why they were included |  |  |
|  |  | (*b*) Report category boundaries when continuous variables were categorized |  |  |
|  |  | (*c*) If relevant, consider translating estimates of relative risk into absolute risk for a meaningful time period |  |  |

Continued on next page

| Other analyses | 17 | Report other analyses done—eg analyses of subgroups and interactions, and sensitivity analyses | 19-20 | as specific details in the manuscript Page 19-20 |
| --- | --- | --- | --- | --- |
| Discussion | | | | |
| Key results | 18 | Summarise key results with reference to study objectives | 20-23 | as specific details in the manuscript Page 20-23(Conclusion and Discussion) |
| Limitations | 19 | Discuss limitations of the study, taking into account sources of potential bias or imprecision. Discuss both direction and magnitude of any potential bias | 25 | Limitations and future research directions |
| Interpretation | 20 | Give a cautious overall interpretation of results considering objectives, limitations, multiplicity of analyses, results from similar studies, and other relevant evidence | 21-23 | as specific details in the manuscript Page 21-23(Discussion) |
| Generalisability | 21 | Discuss the generalisability (external validity) of the study results | 21-23 | as specific details in the manuscript Page 21-23(Discussion) |
| Other information | |  | | |
| Funding | 22 | Give the source of funding and the role of the funders for the present study and, if applicable, for the original study on which the present article is based |  | This work was supported by the Hengshui University High-level Talents Research Initiation Fund Project: “Research on the Influence of High-Performance Human Resource Practices on Physical Education Teachers’ Turnover Intentions in Regional Universities in Hebei Province” (Project Number: 2023GC016);  the 2023 Western Area Project of National Social Science Foundation of China: “Study on Pressure Test of High-level Institutionalized Openness in Pilot Free Trade Zone (Port)” (Project Number: 23XJY023);  Scientific Research Project of Hengshui Universit（2024GC07） |

*Give information separately for cases and controls in case-control studies and, if applicable, for exposed and unexposed groups in cohort and cross-sectional studies.

**Note:** An Explanation and Elaboration article discusses each checklist item and gives methodological background and published examples of transparent reporting. The STROBE checklist is best used in conjunction with this article (freely available on the Web sites of PLoS Medicine at http://www.plosmedicine.org/, Annals of Internal Medicine at http://www.annals.org/, and Epidemiology at http://www.epidem.com/). Information on the STROBE Initiative is available at www.strobe-statement.org.
